# Coal, Gas, and Electricity for Cooking

**Published:** 1913-01-11

**Authors:** 


### PRACTICAL POINTS.

(Criticism and Suggestions Invited.)

### Coal, Gas, and Electricity for Cooking.

Hospitals and other public institutions have now at their command a very wide selection of apparatus for cooking, both on a large and small scale. There is a very keen competition between the makers of apparatus employing coal, gas, and electricity respectively. Electricity has only recently come into the field, but it is making steady headway.

It is well known that it was the threatened competition of electricity for lighting purposes in 1882, at the time when the first electrical exhibition was held at the Crystal Palace, that caused gas engineers to study seriously the problem of heating by gas. In view of the possibility of the lighting business being lost, or even a certain portion of it, they determined to make an effort to secure a portion of the heating business; and they have met with very marked success. As usual, the competition of gas cooking and heating appliances has stimulated the manufacturers of cooking and heating appliances using coal, and coal-consuming apparatus on the market, suitable for the use of hospitals and similar institutions, leave very little to be desired. They are very economical in fuel, they are very cleanly, and by the use of steam, which is freely employed, results have been produced that would have been undreamed of in pre-gas-competition days.

Gas engineers, however, have closely followed the lead of the makers of coal-burning appliances, and have produced almost identical apparatus, in which all that can be done by the use of coal is done by the use of gas, even to the employment of steam for a portion of the cooking, such as for vegetables, and for cooking certain joints; with the added advantage that the gas is brought right to the kitchen in a pipe. The gas is always there, providing the gas pipes and the gas service are in order. There is no question of carrying fuel from storage in the basement to the upper floor, where the architect of the modern hospital appropriately locates his kitchen. In frosty weather it sometimes happens that gas is wanting, because gas engineers have not yet succeeded in eliminating naphthalene from the gas. So long as the atmospheric temperature is above a certain figure naphthalene gives no trouble; but in frosty weather it solidifies and deposits in the pipes, with the result that the gas is throttled, and in many cases the supply is wholly or partially cut off. Gas engineers are fully alive to the trouble from naphthalene; and in a recent issue of the leading journal of the gas industry a method was suggested of getting rid of the trouble entirely. It is more than probable that in the near future naphthalene will be eliminated, and then the supply will be practically certain. Gas also is becoming cheaper. Gas engineers, warned by the threatened competition of electricity in 1882, have been constantly on the look-out for other sources of income, besides the sale of gas; and in recent years nearly all of them have converted what were previously their waste products into the valuable artificial manure, sulphate of ammonia.

### The Competition of Electricity.

A few years ago the idea of electricity seriously competing with gas was looked upon as absurd. Electrical engineers claimed that the whole of the heat they delivered to any apparatus was employed in the purpose for which the apparatus was designed, say, in boiling water, cooking a joint, and so on; but the cost of the heat units delivered to the kettle or to the oven was so enormously in excess of the cost of the same number of heat units delivered by gas or by coal that electricity was practically out of court. As time has gone on, however, and as experience has been made with electrical cooking appliances, two things have happened. Electrical engineers have discovered that the whole of the heat units delivered by their heating elements are not usefully applied, as they supposed, in boiling the water, or cooking the joint, etc. In America, which leads the way in this as in certain other electrical developments, efficiencies of 50 per cent. and 60 per cent. are talked of as being in advance of efficiencies previously obtained. An efficiency of 50 per cent. means that half the heat units delivered to the heating element by the electric current are wasted. There is, of course, a greater loss with gas, because in nearly every gas apparatus a very large portion of the heat that is liberated by the combustion of the gas has to be carried off up the chimney. In gas ovens, for instance, it is absolutely necessary to have a good draught to carry off the production of combustion, and the draught also carries off a large proportion of heat units.

As in so many other developments of electricity, cases have arisen where, notwithstanding the heavy cost of the heat units delivered by the electric current, the method of heating has been economical for other reasons. The electrically-heated kettle standing upon a neat tripod in a lady's drawing-room, for instance, is a striking case in point. The cost of heating the water for afternoon tea is probably many times the cost at which it could be heated by gas, but the actual cost is so small compared with the cost of preparing afternoon tea as a whole that the difference between the gas and electricity does not matter, while the great convenience of having the kettle standing out on the tripod in the drawing-room is worth far more than that difference. There have been special cases also where the cleanliness and convenience of cooking by electricity have been of such great advantage that it has paid the users of the apparatus to employ electricity, notwithstanding its higher cost. There is at least one electrically-worked restaurant in the West of London, whose proprietor states that the cost of running his establishment is less by electricity than by either gas or coal; while the greater cleanliness and convenience would have been worth a good deal to him, even if the cost had been higher. Probably these features will appeal strongly to hospital superintendents. With electricity also there should be no danger of a stoppage of supply in frosty weather, such as there still is with gas. Further, it may be mentioned that manufacturers of electrical cooking and heating appliances have closely copied the apparatus using both coal and gas, even to the provision of steam for carrying out a portion of the cooking. It should be mentioned also that electrical engineers who are running electricity generating stations, and makers of electrical cooking appliances, claim that there is a saving in the meat cooked by electricity over that cooked by gas or coal ovens. It will be remembered that in all cooking there is a certain amount of waste; the finished product weighs somewhat less than the raw. It used to be the practice to allow a wastage of 50 per cent. It is claimed by electrical engineers that there is at least 10 per cent. less waste with electrical cooking appliances than with gas. Gas engineers stoutly deny this, however.

